# Rapid Development of Microsatellite Markers for *Callosobruchus chinensis* Using Illumina Paired-End Sequencing

**DOI:** 10.1371/journal.pone.0095458

**Published:** 2014-05-16

**Authors:** Can-xing Duan, Dan-dan Li, Su-li Sun, Xiao-ming Wang, Zhen-dong Zhu

**Affiliations:** The National Key Facility for Crop Gene Resources and Genetic Improvement, Institute of Crop Science, Chinese Academy of Agricultural Sciences, Beijing, People’s Republic of China; Kansas State University, United States of America

## Abstract

**Background:**

The adzuki bean weevil, *Callosobruchus chinensis* L., is one of the most destructive pests of stored legume seeds such as mungbean, cowpea, and adzuki bean, which usually cause considerable loss in the quantity and quality of stored seeds during transportation and storage. However, a lack of genetic information of this pest results in a series of genetic questions remain largely unknown, including population genetic structure, kinship, biotype abundance, and so on. Co-dominant microsatellite markers offer a great resolving power to determine these events. Here, we report rapid microsatellite isolation from *C. chinensis* via high-throughput sequencing.

**Principal Findings:**

In this study, 94,560,852 quality-filtered and trimmed reads were obtained for the assembly of genome using Illumina paired-end sequencing technology. In total, the genome with total length of 497,124,785 bp, comprising 403,113 high quality contigs was generated with *de novo* assembly. More than 6800 SSR loci were detected and a suit of 6303 primer pair sequences were designed and 500 of them were randomly selected for validation. Of these, 196 pair of primers, i.e. 39.2%, produced reproducible amplicons that were polymorphic among 8 *C. chinensis* genotypes collected from different geographical regions. Twenty out of 196 polymorphic SSR markers were used to analyze the genetic diversity of 18 *C. chinensis* populations. The results showed the twenty SSR loci were highly polymorphic among these populations.

**Conclusions:**

This study presents a first report of genome sequencing and *de novo* assembly for *C. chinensis* and demonstrates the feasibility of generating a large scale of sequence information and SSR loci isolation by Illumina paired-end sequencing. Our results provide a valuable resource for *C. chinensis* research. These novel markers are valuable for future genetic mapping, trait association, genetic structure and kinship among *C. chinensis*.

## Introduction

The adzuki bean weevil, *Callosobruchus chinensis* (L.), is one of the most destructive pests of leguminous stored seeds in Asia. *C. chinensis* larvae utilize a variety of dried legume seeds as their hosts, primarily *Vigna* species and genera such as *Cajanus* and *Lens* [1], [2]. This insect often causes considerable damage to stored mungbean (*Vigna radiata* L.), cowpea (*Vigna unguiculata* L.), adzuki bean (*Vigna angularis* Ohwi & Ohashi), faba bean (*Vicia faba* L.), pea (*Pisum sativum* L.), chickpea (*Cicer arietinum* L.), soybean (*Glycine max* L. Merr.) and lotus seed (*Nelumbo nucifere* Gaertn.). Usually, this beetle causes 32∼64% *Vigna* seeds loss during transportation and storage and infestation rate may reach 100% with 3–5 months of storage [3], [4], which results in loss of mass and low germination rate and makes them unfit for human consumption or for agricultural and commercial use [5].

Microsatellites or simple sequence repeats (SSRs) are tandemly repeated motifs of one to six bases found in the nuclear genomes of all eukaryotes tested and are often abundant and evenly dispersed [6]–[8]. Microsatellite sequences are usually characterized by a high degree of length polymorphism, and are ideal single-locus co-dominant markers for genetical studies. Microsatellites are the most frequently used DNA markers in many areas of research because of their high abundance, multi-allelic nature, high polymorphism rates, and rapid detection of the alleles by wide variety of methods [9]–[11].

However, their availability and quality are limited by the difficulties of *de novo* development in organisms without available genomic information. Up to date, none of SSR loci as well as other makers such as AFLP, RAPD and AFLP in *C. chinensis* has been isolated and identified, which results in a large number of genetic information related to *C. chinensis* has not been revealed. The most commonly used procedure for microsatellite isolation is enrichment of genomic DNA in microsatellite motifs, followed by cloning and sequencing of the enriched DNA library by the Sanger method, which is difficult, time-consuming and costly. Enrichment methods use a few specific repeated motifs, generally selected without prior knowledge of their abundance in the genome [12], hence introducing potential bias in genome representativeness.

Recently developed next-generation sequencing (NGS) platforms such as SOLiD (ABI, Norwalk, CT), 454 GS FLX (Roche, Penzberg, Germany), and Illumina Genome Analyzer (Illumina, San Diego, CA) can facilitate high-throughput genome sequencing of both noncoding and coding regions, including large scale resequencing in well-characterized species or *de novo* transcriptome sequencing for species without reference sequences. These technologies are capable of generating a large number of reads at a relatively low cost. The huge amounts of sequence data produced on these high-throughput platforms can be used to identify a great quantity of genetic markers [13]–[17].

In the past few years, the 454 pyrosequencing has been preferred for non-model organisms [18]–[21] due to its longer read length than Illumina sequencing. With shorter read length, Illumina platform was at first confined to re-sequencing applications which rely on a reference sequence. Recently, with increased read length by Illumina technology improvement and development of new computational tools, short reads can be assembled and used for transcriptome or genome analysis. Illumina sequences now can produce moderately long reads (up to 150 bp with the GAIIx, and 100 bp with the HiSeq 2000) and accommodate paired-end sequencing from both ends of ∼200–600 bp fragments. There have also been massive increases in the number of reads obtained per Illumina sequencing run. Furthermore, compared with relatively constant cost of 454 sequencing, the cost of obtaining Illumina sequence data has dropped substantially [22]. *De novo* assemblies using sequence reads from Illumina technology have been reported in many non-model organisms without a reference sequence [23]–[27]. To take advantage of these advances, we utilized Illumina paired-end sequencing to isolate SSR loci rapidly and massively.

To the best of our knowledge, this is the first systematic report on development of microsatellite markers of adzuki bean weevil with *de novo* assembly using Illumina paired-end sequencing. The present study identified a large scale of putative SSR loci efficiently and inexpensively using Illumina HiSeq 2500 platform. The resulting SSR sequences were characterized and validated through successful amplification of randomly selected target loci across a selection of adzuki bean weevil genotypes from diverse geographic regions.

## Materials and Methods

### Insect Material

The adzuki bean weevil male adults for genome sequencing were collected from infected mungbean stored at the Institute of Crop Science (ICS), Chinese Academy of Agricultural Sciences (CAAS), Beijing. The *C. chinensis* genotypes for polymorphism detection and genetic diversity were collected from eight and eighteen different regions, respectively (Table 1 and Table 2).

**Table 1:** The information of 8 *Callosobruchus chinensis* genotypes used in polymorphism test of SSR markers.

Populationcode	Number ofindividuals	Occurrence location	Collectiontime	Host
DZ	30	Dengzhou, Henan	2012	mungbean
FC	30	Fuchuan, Guangxi	2012	mungbean
HH	30	Hohhot, Inner Mongolia	2012	mungbean
MT	30	Mengtougou, Beijing	2013	adzuki bean
QD	30	Qingdao, Shandong	2013	mungbean
TS	30	Tangshan, Hebei	2013	adzuki bean
UM	30	Urumuqi, Xinjiang	2012	mungbean
WL	30	Wulong, Chongqing	2012	mungbean

**Table 2:** The information of 18 *Callosobruchus chinensis* populations used in genetic diversity analysis.

Populationcode	Number ofindividuals	Occurrence location	Collectiontime	Host
BB	20	Bobai, Guangxi	2012	mungbean
BD	20	Baoding, Hebei	2013	adzuki bean
DX	20	Daxin, Guangxi	2012	mungbean
DZ	20	Dengzhou, Henan	2012	mungbean
HF	20	Hefei, Anhui	2012	mungbean
HH	20	Hohhot, Inner Mongolia	2012	mungbean
JC	20	Jiangchuan, Yunnan	2012	mungbean
LC	20	Lingchuan, Shanxi	2013	adzuki bean
LL	20	Luliang, Yunnan	2012	mungbean
LS	20	Lishui, Jiangsu	2013	adzuki bean
MC	20	Mengcheng, Anhui	2012	mungbean
QD	20	Qingdao, Shandong	2013	mungbean
QJ	20	Qianjiang, Chongqing	2012	mungbean
RD	20	Rudong, Jiangsu	2012	adzuki bean
TS	20	Tangshan, Hebei	2013	adzuki bean
UM	20	Urumuqi, Xinjiang	2012	mungbean
XY	20	Xinye, Henan	2012	adzuki bean
YY	20	Yangyuan, Hebei	2013	mungbean

### DNA Isolation, Quantification and Qualification

Total genomic DNA of single male adult with belly removal was extracted with TIANamp Genomic DNA Kit according to the manufacturer’s instructions (TIANGEN, Beijing, China). The quality of extracted DNA was monitored on 1.5% agarose gels. DNA purity was checked using the NanoPhotometer spectrophotometer (IMPLEN, San Diego, CA). DNA concentration was measured using Qubit DNA Assay Kit in Qubit 2.0 Flurometer (Life Technologies, San Diego, CA). Fragment distribution of DNA library was measured using the DNA Nano 6000 Assay Kit of Agilent Bioanalyzer 2100 system (Agilent Technologies, San Diego, CA).

### DNA Library Preparation for Sequencing

A total amount of 3 µg genomic DNA per sample was used as input material for the DNA sample preparation. Sequencing libraries were generated using Illumina Truseq DNA Sample Preparation Kit (Illumina, San Diego, CA) following manufacturer’s recommendations and x index codes were added to attribute sequences to each sample. Briefly, fragmentation was carried out by hydrodynamic shearing system (Covaris, Woburn, MA) to generate <800 bp fragments. Remaining overhangs were converted into blunt ends via exonuclease/polymerase activities and enzymes were removed. After adenylation of 3′ ends of DNA fragments, Illumina PE adapter oligonucleotides were ligated to prepare for hybridization. In order to select DNA fragments of preferentially 500 bp in length, agarose electrophoresis was performed (120V, 40 min, 1.5% agarose) and adapter-ligated constructs derived from 620 bp to 670 bp was separated. After purification using spin column (QIAGEN, Dusseldorf, Germany), DNA fragments with ligated adapter molecules on both ends were selectively enriched using Illumina PCR Primer (F: 5′-AATGATACGGCGACCACCGAGA-3′ and R: 5′-CAAGCAGAAGACGGCATACGAGT-3′) Cocktail in a PCR reaction with 10 cycles. Products were purified using AMPure XP system (Beckman Coulter, Beverly, MA) and quantified using the Agilent high sensitivity DNA assay on the Agilent Bioanalyzer 2100 system.

### Clustering and Sequencing

The clustering of the index-coded samples was performed on a cBot Cluster Generation System using TruSeq PE Cluster Kit v3-cBot-HS (Illumia, San Diego, CA) according to the manufacturer’s instructions. After cluster generation, the library preparations were sequenced on an Illumina Hiseq 2500 PE150 platform (Illumina, San Diego, CA) and 150 bp paired-end reads were generated. The sequencing data have been submitted to the National Center for Biotechnology Information (NCBI) short read archive (SRA) and given the accession numbers SRR949786 and SRR952345.

### Data Filtering, *De Novo* Assembly and Decontamination

Before the genome assembly, we carried out a stringent filtering process of raw sequencing reads. The reads with more than 10% of bases with a quality score of Q<20, or the paired reads if the N content of a single read exceeds 10% of the read length, ambiguous sequences represented as “N” and adaptor contamination were removed. *De novo* genome assembly was performed by de Bruijn graph and SOAPdenovo [28] software package with the default settings except K-mer value. The high-quality reads were loaded into the computer, and then de Bruijn graph data structure was used to represent the overlap among the reads. After adjusting for various parameters, an 87-mer assembly was finally selected. In order to ensure the accuracy and validity of the SSR search, the contigs less than 500 bp length were filtered out.

In view of the fact that some *Wolbachia* strains are the endosymbionts living in the cytoplasm of bean beetles, such as *C. chinensis* and *Callosobruchus analis* [29], [30], the sequencing data of *C. chinensis* were likely to be contaminated with *Wolbachia*’s sequences, hence decontamination had to be performed. First, re-assembly should be performed after removal of the *Wolbachia* sequences from the valid sequencing data. Specifically, based on the distribution plot of contig depth and GC content, contigs with a depth greater than 50× (contamination contigs) were extracted and cut into the 87-mer fragments. The fragments were aligned with the valid sequence data and the matched reads were removed. Second, the reassembled *C. chinensis* genome was aligned with records in bacterial and fungal genome databases and the matched contigs were also removed.

### SSR Loci Search and Primer Design

Potential SSR markers were detected among the assembled genome using the MISA tool [31] (http://pjrc.ipk-gatersleben.de/misa/). We searched for SSRs with motifs ranging from monoto hexa-nucleotides in size. The minimum of repeat units were set as follows: ten repeat units for mono-nucleotide, six for dinucleotides and five for tri-, tetra-, penta- and hexa-nucelotides. Mono-nucleotide repeats were discarded in that it was difficult to distinguish genuine mono-nucleotide repeats from polyadenylation products and some mono-nucleotide repeats were generated by base mismatch or sequencing errors. Primer pairs were designed using Primer3 [32] (http://fokker.wi.mit.edu/primer3/) with default parameters.

### SSR Marker Polymorphism Detection and Assessment

Five hundred randomly selected primer pairs were synthesized (Sangon Biotech, Beijing, China) for polymorphism detection among 8 *C. chinensis* genotypes (Table 1). Polymerase chain reactions (PCR) were carried out in 10 µL reaction volumes containing 10 mmol/L Tris-HCl pH 8.3, 50 mmol/L KCl, 1.5 mM MgCl, 250 mM each of dNTPs, 0.2 M of each primers, 0.5 U Taq polymerase (Dingguo, Beijing, China) and 30 ng of DNA template. Reactions were performed using a GeneAmp PCR System 9700 thermal cycler (ABI, Norwalk, CT) programmed as 94°C for 4 min, followed by 35 cycles of 94°C for 30 s, 50–60°C (it depends optimized annealing temperature) for 30 s and 72°C for 60 s. The final extension was performed at 72°C for 10 min. The PCR products were analyzed by electrophoresis on 8.0% non-denaturing polyacrylamide gels and silver stained [33]. The band sizes were determined roughly based on 100 bp DNA ladder (TIANGEN, Beijing, China).

The genotyping data was subsequently used to determine genetic relationships among 18 diverse *C. chinensis* populations collected from different regions (Table 2). The number of alleles (*Na*), expected heterozygosity (*He*), observed heterozygosity (*Ho*) and Shannon’s information index (*I*) were calculated using POPGEN1.32 software [34], [35]. Using the MEGA 4 software, based on Nei’s genetic distance, a phylogenetic tree was constructed using the neighbor-joining clustering method [36].

## Results

### Illumina Sequencing and Quality Control

The paired-end sequencing yielded 2×150-bp reads from either end of the DNA fragment. In this study, a total of 32 Gb of raw sequencing data, comprising 106,888,024 raw sequencing reads were generated from a 300 bp insert library. After stringent quality assessment and data filtering, 94,560,852 (88.47%) clean reads with Q20 bases (those with a base quality greater than 20) were deemed as high quality reads and used for further analysis. The sequences have been deposited in NCBI. Output data are shown in Table 3, in which Bpis2 was an additional sequencing data, indicating it was a high-quality (Q20≥96%, Q30≥91%) sequencing and had a normal sequencing error rate (0.04–0.06%) (Figure S1 and Figure S2).

**Table 3:** Summary of adzuki bean weevil sequencing data output.

Samples ID	Rawreads	Effectiverate (%)	Clearreads	Q20 (%)	Q30 (%)	GCContent (%)
Bpis1	52,566,784	88.64	46,595,197	98.81; 96.42	96.38; 91.83	36.76; 36.75
Bpis2	54,321,240	88.30	47,965,654	98.73; 96.27	96.16; 91.47	36.75; 36.74

Notes: Effective rate (%) = clear reads/raw reads×100, Q20 and Q30 refer to the values of quality of sequencing data.

In order to detect whether the sequences were contaminated by endosymbiont sequences, part of the valid sequencing data were analyzed by BLAST against NCBI nucleotide database (NT) and found that 1.54% and 1.57% of the sequencing data were associated with the genus *Wolbachia*, respectively, indicating that bacterial contamination was present in the samples. Therefore, decontamination has to be performed in order for veritable assembly of the *C. chinensis* genome.

### Genome *De Novo* Assembly and Decontamination

The 94,560,852 high-quality clean reads were used to assemble the genome of *C. chinensis* with SOAPdenovo software. According to the overlapping information of high-quality reads, an 87-mer assembly was finally selected and the assembled *C. chinensis* genome was generated with the total length of 499,585,451 bp, comprising of 405,744 contigs with an average length of 1237 bp, a N50 of 1439 bp, N90 of 608 bp and 36.47% of GC content.

The GC content scatterplot showing only one species will show a concentrated distribution. Taking account of sequencing data probably contained bases from the genus *Wolbachia*, the GC distribution was analyzed in order to determine whether the assembly contained contamination from non-target species or not. In the scatterplot of contigs depth against GC content, the points were divided into three parts (Figure S3). A BLAST analysis of the contigs sequence of each part to the nucleotide database showed that contigs with a depth greater than 50 were contaminated with bacteria.

**Figure 1:**
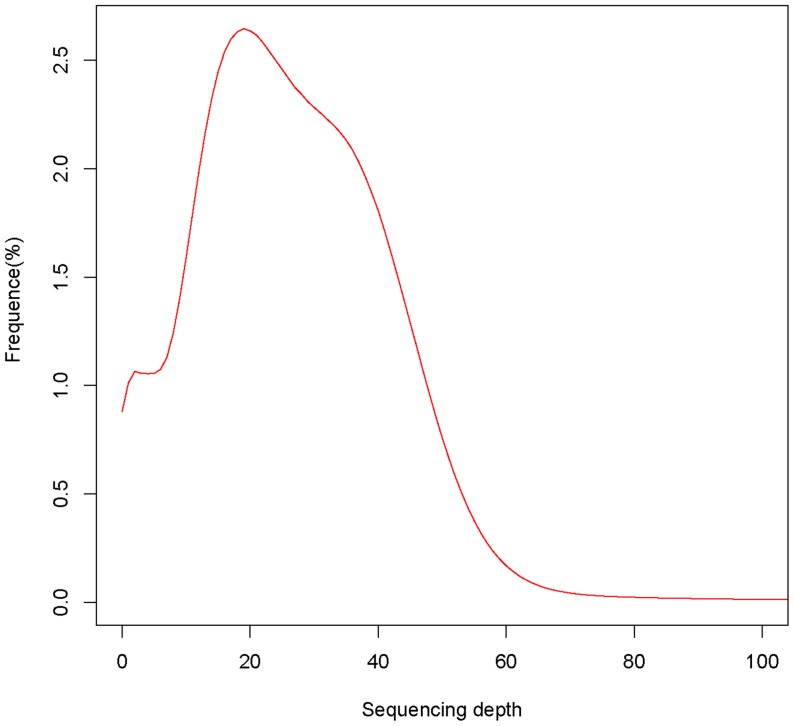
Distribution of sequencing depth.

**Figure 2:**
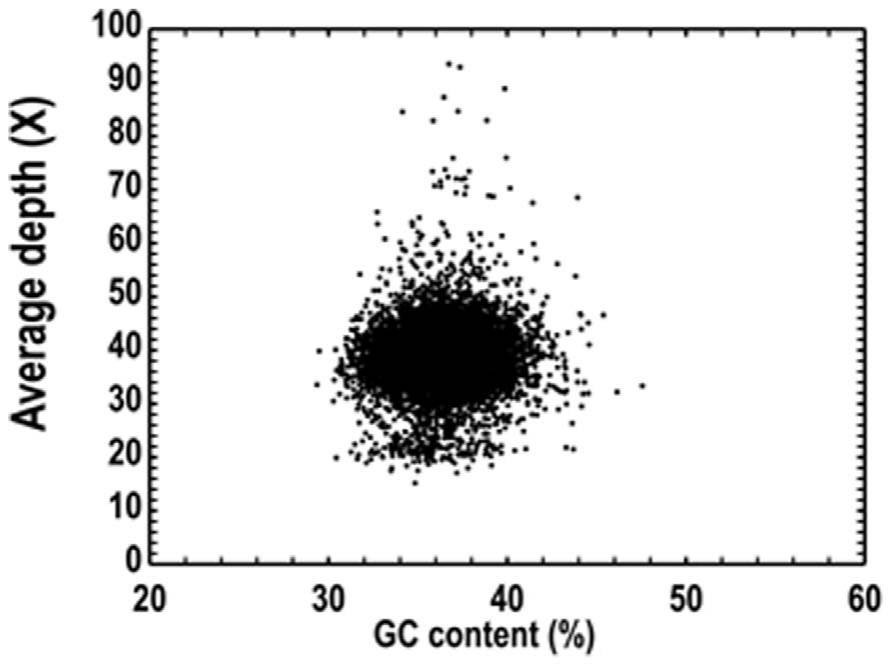
Distribution between GC content and sequencing depth.

To facilitate better and authentic assembly of the *C. chinensis* genome, decontamination analysis was carried out (Table S1 and Table 4). Finally, the assembled *C. chinensis* genome was obtained with the total length of 497,124,785 bp, comprising of 403,113 contigs (Table 5). The Adenine, thymine, cytosine and guanine content and the abundance in *C. chinensis* genome are shown in Table 6. The coverage of contigs assembled with decontaminated clean reads reaches 99.12%, with an average sequencing depth of about 20× (Figure 1). A scatterplot of the sequencing depth against the GC content was generated and the vast majority of the points appeared in a relatively narrow range, indicating the final assembled genome was free of contamination (Figure 2).

**Table 4:** Re-assembly of adzuki bean weevil genome after decontamination.

Sample ID	Totallength(bp)	Contigs number	N50 (bp)	N90 (bp)	GC(%)
Bpis	499,585,451	403,617	1,439	608	36.48

**Table 5:** Final assembly of adzuki bean weevil genome.

Sample ID	Totallength(bp)	Contigsnumber	N50 (bp)	N90 (bp)	GC(%)
Bpis	497,124,785	403,113	1,432	606	36.48

**Table 6:** The base content in adzuki bean weevil genome.

Base Type	Number (bp)	% of Genome
Adenine (A)	157,892,519	31.76
Thymine (T)	157,873,741	31.76
Cytosine (C)	90,693,363	18.24
Guanine (G)	90,665,162	18.24
GC	36.48%	
Total	497,124,785	

### Identification of SSR Loci

**Figure 3:**
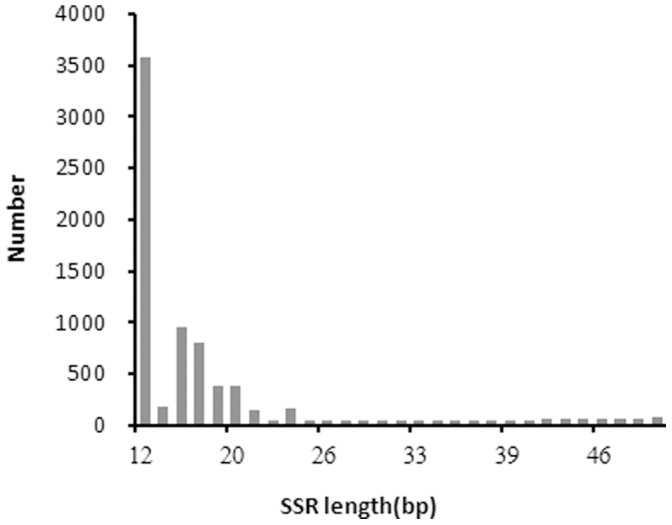
Distribution between SSR length and number of SSRs.

**Figure 4:**
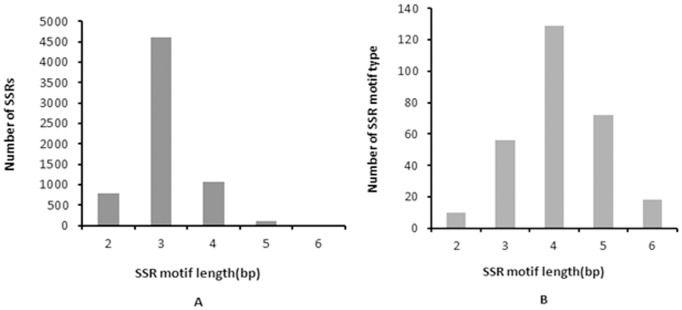
Distribution of SSR motif. (**A**) Distribution between SSR motif and number of SSRs. (**B**) Distribution between SSR motif and number of SSR motif type.

After MISA analysis, a total of 6,593 potential simple sequence repeats (SSRs) were identified with the length of 12–64 bp. The majority of the SSR sequences were from 12 to 25 bp in length, accounting for 98.62% of the total identified SSR loci (Figure 3). Of these, the most frequently detected SSR sequences in *C. chinensis* consisted of the motif of trinucleotide repeats, with 70.02% of abundance, followed by tetranucleotide (16.35%) and dinucleotide repeats (11.80%) (Figure 4A). The length of SSR motifs ranged from 2 to 6 bp and the most common type of SSR motifs comprised tetranucleotide repeats, followed by pentanucleotide and trinucleotide (Figure 4B). More than 6800 SSR loci were detected and a total of 6,303 primer pairs were designed by Primer3 (Table S2) for future assessment of validity by locus amplification.

### Validation of SSR Assay

**Figure 5:**
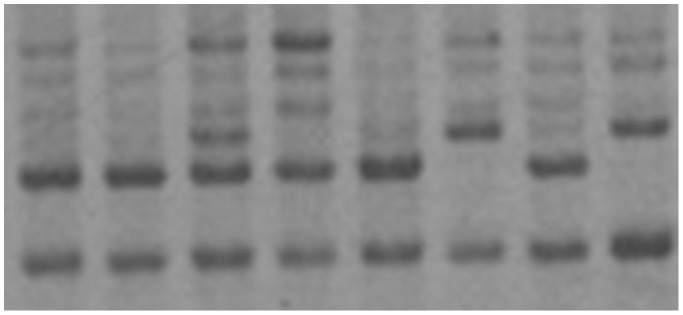
Polymorphism of primer CCM46 among 8 *Callosobruchus chinensis* genotypes.

**Figure 6:**
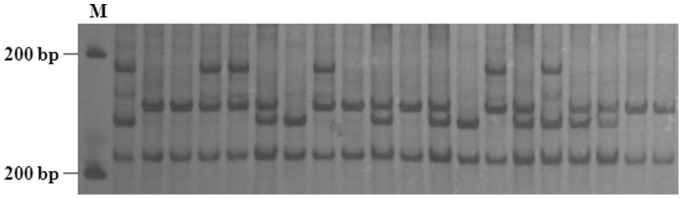
SSR PCR amplification patterns of 20 *Callosobruchus chinensis* individuals from RD population using primer CCM46. (M is the 100 bp DNA ladder marker).

**Figure 7:**
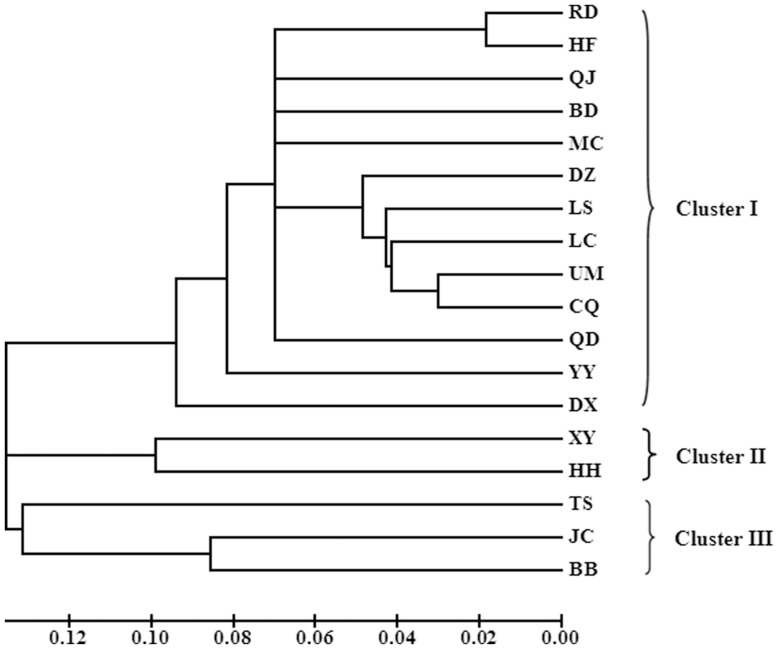
Dendrogram for 18 populations of *Callosobruchus chinensis* in China based on 20 SSR loci (Population code see table 2).

Five hundred primer pairs were randomly selected to evaluate the rate of amplication and polymorphism in 8 diverse *C. chinensis* genotypes, of which 403 pairs (80.6%) successfully amplified clear fragments and 365 pairs (73.0%) produced PCR amplicons with the expected size. Of these, 196 pairs (39.2%) were polymorphic among 8 genotypes of *C. chinensis* (Figure 5 and Table S3). The genetic relationship among 18 diverse *C. chinensis* populations from different regions were performed using 20 polymorphic SSR pairs randomly selected from 196 pairs (Figure 6 and Table 7). The number of alleles (*Na*) per locus ranged from 4 to 13 with an average of 7.35, the expected heterozygosity (*He*) ranged from 0.0591 to 0.7868, and the observed heterozygosity (*Ho*) varied from 0.0075 to 0.6815. Shannon’s information index (*I*) values ranged from 0.1659 to 1.6831 with an average of 0.9545 (Table 8). Based on to Nei’s genetic distance, the dendrogram generated from the cluster analysis showed the genetic relationship among 18 *C. chinensis* populations. As shown in Figure 7, the 18 populations were classified into three distinct genetic groups at the genetic distance of 0.14 or so. The majority of populations were grouped into cluster I (RD, HF, OJ, BD, MC, DZ, LS, LC, UM, CQ, QD, YY and DX), while cluster II and cluster III included merely 2 (XY and HH) and 3 (TS, JC and BB) populations, respectively.

**Table 7:** Characteristics of 20 polymorphic SSR markers used in genetic diversity analysis (F = forward primer, R = reverse primer, Size = size of cloned allele, Ta = annealing temperature).

Primer	F (5′–3′)	R (5′–3′)	Size (bp)	*Ta* (°C)
CCM1	TTGGTGCTAACGTACATGAAATGAA	CTCGCATAACGACTTCATGGATTT	155	58
CCM2	CAGGACTGGACTGGACATGTGATA	TGCTCAGCCTAATTCAACCTGTTT	156	55
CCM19	AGCTATCCTCATAATTGCTCACGG	CCTCCTTATAAGTGCATTTGTTTGC	138	56
CCM25	AAACTCTTGCGTTAATTGTGCCAT	TTTACTTGATGACTTGGCAGAGCC	99	55
CCM27	TGTGTTCTATCTAGCGTGACTGGG	ATGCTTGTACATTTTGACTTGCCA	158	55
CCM29	CAATGGCATGTCCACCACTACT	GATAAGCAATTGCCTCTTATCCCA	146	55
CCM40	AGGGGATATCGTGTTCTCTGTTCA	GATCGAACCTCGAAGCAGAAGTT	115	55
CCM46	AACGGACAGACAGAGAGATAGTCAA	TCTGTTTGTTTGCCTGTTTGC	134	55
CCM49	GTTGACCGTTGGACAATAAAGCTA	TGTCACTACAATCGATTCGTCTCAT	145	55
CCM56	CCTAAGAGTTTCGTTTTTGCATGG	GGCAAATTGAGCTCAGCATTAGAT	110	55
CCM65	GTACCGCGGGTACCTACGTACTTT	GTGTTTACCGTTTTCAAACGCAG	81	55
CCM66	GGGCTAATCGCACAACTGTAAATC	AAATAGCCAGGAGCTAGACTTCGC	141	58
CCM83	AGGGTTTTTCCCGTTTAACGATATT	CGGATCTGGAAGCAGTTTTGTTTA	140	57
CCM108	ACTTAAATTACCCGGTGACGTGAA	AACTTTTGTAAATCGCGCTCAAAG	131	55
CCM118	TTAATTCTGGTCCTGTTCCGTTTT	TCTTCTAATCCCATGACTAGCTTCAC	130	55
CCM125	AGCGTGTTAAAAAGAGAGTGGACG	TATTATTCTGCAGATGTGCCGCT	144	57
CCM149	CTTTGCAGTTACGAGAGACAGGGT	TGCCAACCTTCTATAAGAACACGG	139	57
CCM154	TTGATCCTTTTCCTCCCAATAAAA	TTCGTCCTCATATAGCACAAATGTT	145	53
CCM180	TACAACTGTAAATGGCGTTTGGCT	GGCATTTCAACAAAAGTACGCTTC	116	55
CCM189	GGAAGTTGCATATGTGAAGTCCAA	AGGATGCATTCTGTGTCATCTGTT	107	56

**Table 8:** Informativeness of SSR loci following amplification from 18 geographically diverse *Callosobruchus chinensis* populations in China.

Locus	*Na*	*He*	*Ho*	*I*
CCM1	9.0000	0.7455	0.2171	1.5977
CCM2	6.0000	0.0801	0.0597	0.2283
CCM19	4.0000	0.2564	0.2340	0.5288
CCM25	8.0000	0.5384	0.1929	1.0222
CCM27	13.0000	0.4222	0.2587	0.8954
CCM29	4.0000	0.3424	0.3885	0.6624
CCM40	6.0000	0.4502	0.3372	0.9405
CCM46	7.0000	0.6956	0.5059	1.4290
CCM49	7.0000	0.5437	0.5075	0.9823
CCM56	13.0000	0.6890	0.6815	1.5153
CCM65	4.0000	0.5212	0.3583	0.8078
CCM66	6.0000	0.2339	0.0800	0.5297
CCM83	9.0000	0.3247	0.2939	0.6864
CCM108	8.0000	0.5260	0.3360	0.9079
CCM118	9.0000	0.7868	0.3386	1.6831
CCM125	7.0000	0.4158	0.0075	0.7910
CCM149	5.0000	0.0591	0.0361	0.1659
CCM154	6.0000	0.6179	0.3431	1.1863
CCM180	9.0000	0.7292	0.4826	1.4833
CCM189	7.0000	0.5422	0.3034	1.0475

Notes: Number of alleles (*Na*), expected heterozygosity (*He*), observed heterozygosity (*Ho*) and Shannon’s information index (*I*).

## Discussion

This study demonstrated that Illumina paired-end sequencing is capable of identifying massive numbers of potentially PCR-amplifiable SSR loci with relative low-cost. We find that on a read-by-read basis, Illumina paired-end sequences are effective for developing SSR markers in non-model organism such as *C. chinensis*. Compared with Roche GS FLX, the Illumina platform was originally utilized in the organisms with reference genomes [37]–[40]. With the rapid development of various NGS technologies along with a series of novel assembly methods in recent years, *de novo* sequencing and assembly of genome or transcriptome have been successfully used for model [41], [42] and non-model organisms [43]–[46]. Consistent with previous reports, the results from this research also suggested that short reads from Illumina sequencing can be effectively assembled and used for SSR marker development or gene identification in non-model organisms. In this study, more than 94 million high-quality clean reads were used to assemble the genome of *C. chinensis*. This large dataset resulted in a sequencing depth with an average of 20 folds. Finally, the adzuki bean weevil genome with length of 497,124,785 bp was obtained, comprising 403,113 contigs by means of quality-filtering, assembly, decontamination and reassembly.

No molecular maker is available in *C. chinensis* to date. In the previous research, based on the sequence variations of mitochondrial DNA (mtDNA), cytochrome coxidase I gene (COI), and the second internal transcribed spacer of nuclear ribosomal DNA (rDNA ITS2) rather than molecular marker, genetic relationship among *C. chinensis* populations was analyzed [47], [48], which was relatively time-consuming, costly and inconvenient. Nowadays, polymorphic SSR markers play an important role in genetic diversity research, linkage map construction, identification of varieties, comparative genomics and related analysis. The genome sequencing and assembly provided plenty of sequences for developing numerous markers in *C. chinensis*. We performed a general screen on *C. chinensis* genome for the presence of microsatellites. Based on the *de novo* assembled genome, a total of 6,593 potential SSRs were isolated and identified with the most common SSR motifs of trinucleotide, tetranucleotide and dinucleotide repeats. The majority of the SSR sequences ranged from 12 to 25 bp in length, which was reasonable and similar to other reports [49], [50]. Given the types of SSR motifs and the large number of SSR loci identified in our study, future SSR marker isolation may be preferentially focused on those composing of trinucleotide repeats which have been demonstrated to be highly polymorphic and stably inherited in the human genome [51]–[53]. The tri-, tetra-, and dinucleotide repeats mostly contributed to the major proportion of SSRs in this study, while a very small share was contributed by penta- and hexa-nucleotide repeats.

In this study, 6,303 SSR markers were developed and a total of 500 primer pairs were used for assessment of the assembly quality and validity of markers. Of these, 365 pairs produced PCR amplicons with the expected size and 196 pairs were polymorphic among 8 *C. chinensis* genotypes. Most of the SSR primers generated high quality amplicons, suggesting that the genome sequencing and assembly were accurate, and SSR makers are valid and applicable.

Genetic diversity analysis among 18 different geologic populations based on 20 polymorphic SSR loci confirmed validity and usability of polymorphic SSR markers (Table 7). The average number of alleles and Shannon’s information index were 7.35 and 0.9545, respectively among the 20 microsatellite loci indicating that these primers provided abundant polymorphic information in 18 populations and uniform distribution of allele frequencies. Hence the genetic diversity analysis based on these SSR markers is reliable and informative. Gene heterozygosity is considered to be an optimum parameter to measure genetic variation within populations [54]. The average expected heterozygosity was 0.4760, suggesting moderate genetic diversity among the samples, which was likely to be caused by their high adaptability and frequent communication. Based on 20 polymorphic SSR loci, genetic relationship among 18 populations was clearly shown in dendrogram graph in spite that the level of genetic structure variation among populations was modest (Figure 7), indicating relatively high resolution power of SSR makers and applicability in phylogenetics.

All in all, development of SSR markers is useful to elucidate the population genetic structure, and to monitor migration and biotype abundance among adzuki bean weevil or sibling species such as cowpea weevil (*Callosobruchus maculatus* Fabricius) populations. In the meantime, these markers could be used for linkage map construction, gene mapping. Furthermore, the assembled genome will be beneficial to further research such as gene identification, annotation, and so on, which will certainly accelerate the research progress in molecular biology and genetics of adzuki bean weevil and find new measures to control it.

## Conclusion

In this work, we reported a major advance in the identification of large numbers of informative SSR loci in *C. chinensis*. This is a great attempt to study genomic information and develop SSR markers of *C. chinensis* without reference genome using Illumina paired-end sequencing technology. Based on the *de novo* assembly genome, we developed 6,303 SSR markers, and some of which were verified their validity among diverse *C. chinensis* genotypes and populations. These results fully demonstrate that Illumina paired-end sequencing is a rapid and cost-effective approach for SSR markers development in non-model organism.

## Supporting Information

Figure S1 **Distribution of sequencing quality (Q20 and Q30 refer to the values of quality of sequencing data).**
(PDF)

Figure S2 **Distribution of sequencing error rate.**
(PDF)

Figure S3 **Distribution of GC content and contig depth.**
(PDF)

Table S1 **Decontamination of valid sequencing data.**
(DOC)

Table S2 **The primer pairs of** ***Callosobruchus chinensis*** **designed successfully by Primer3.**
(XLS)

Table S3 **Characteristics of 196 polymorphic SSR markers developed in** ***Callosobruchus chinensis*** **L.**
(DOC)
