# Supplementary figures and images for: Rapid Development of Microsatellite Markers for Callosobruchus chinensis Using Illumina Paired-End Sequencing
Source: PLoS One. 2014 May 16;9(5):e95458. doi: 10.1371/journal.pone.0095458 (PMC4023940; doi:10.1371/journal.pone.0095458)

**Qualities distribution**

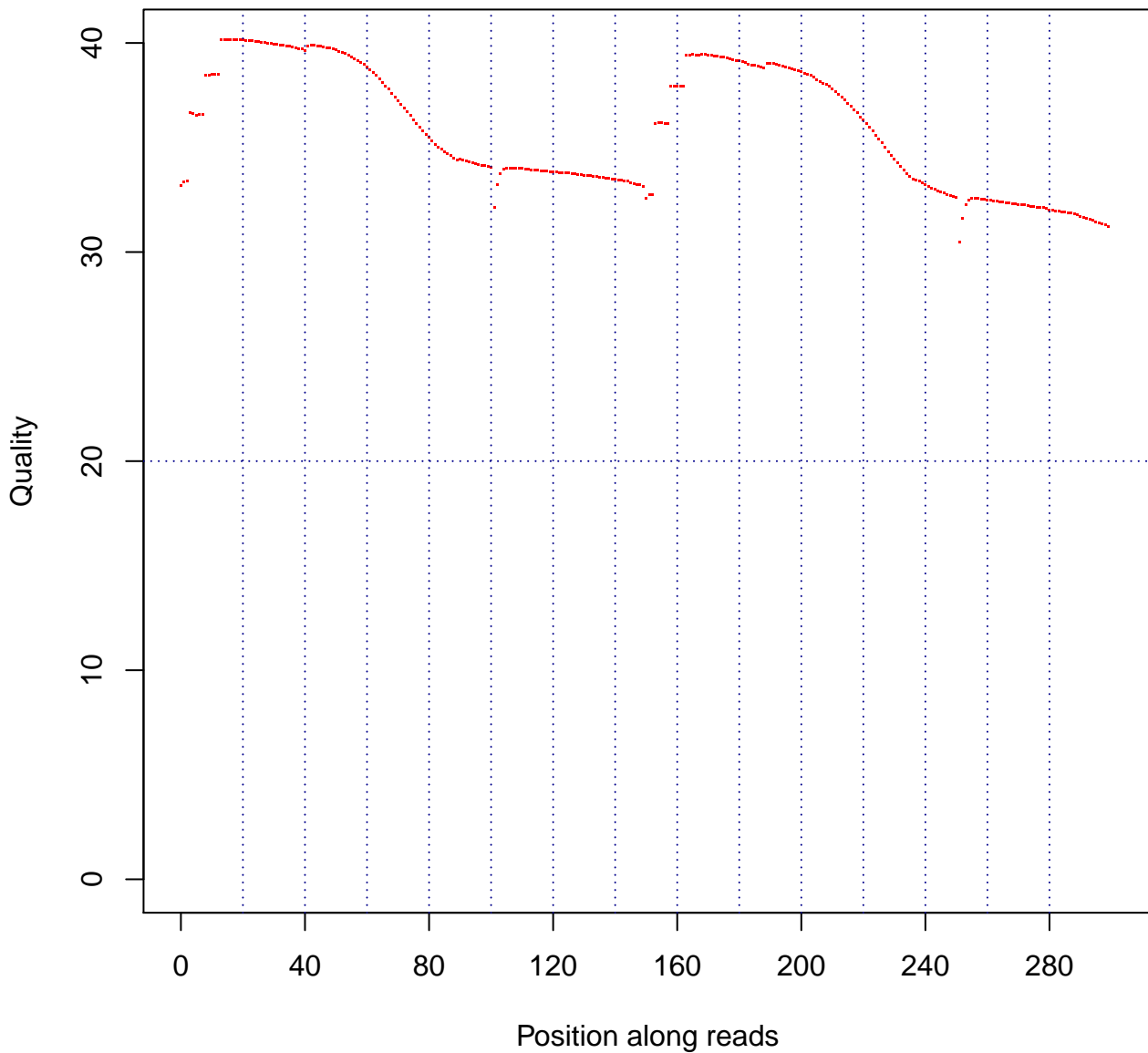

Supplement: Figure S1 — Distribution of sequencing quality (Q20 and Q30 refer to the values of quality of sequencing data). (PDF) [file pone.0095458.s001.pdf]

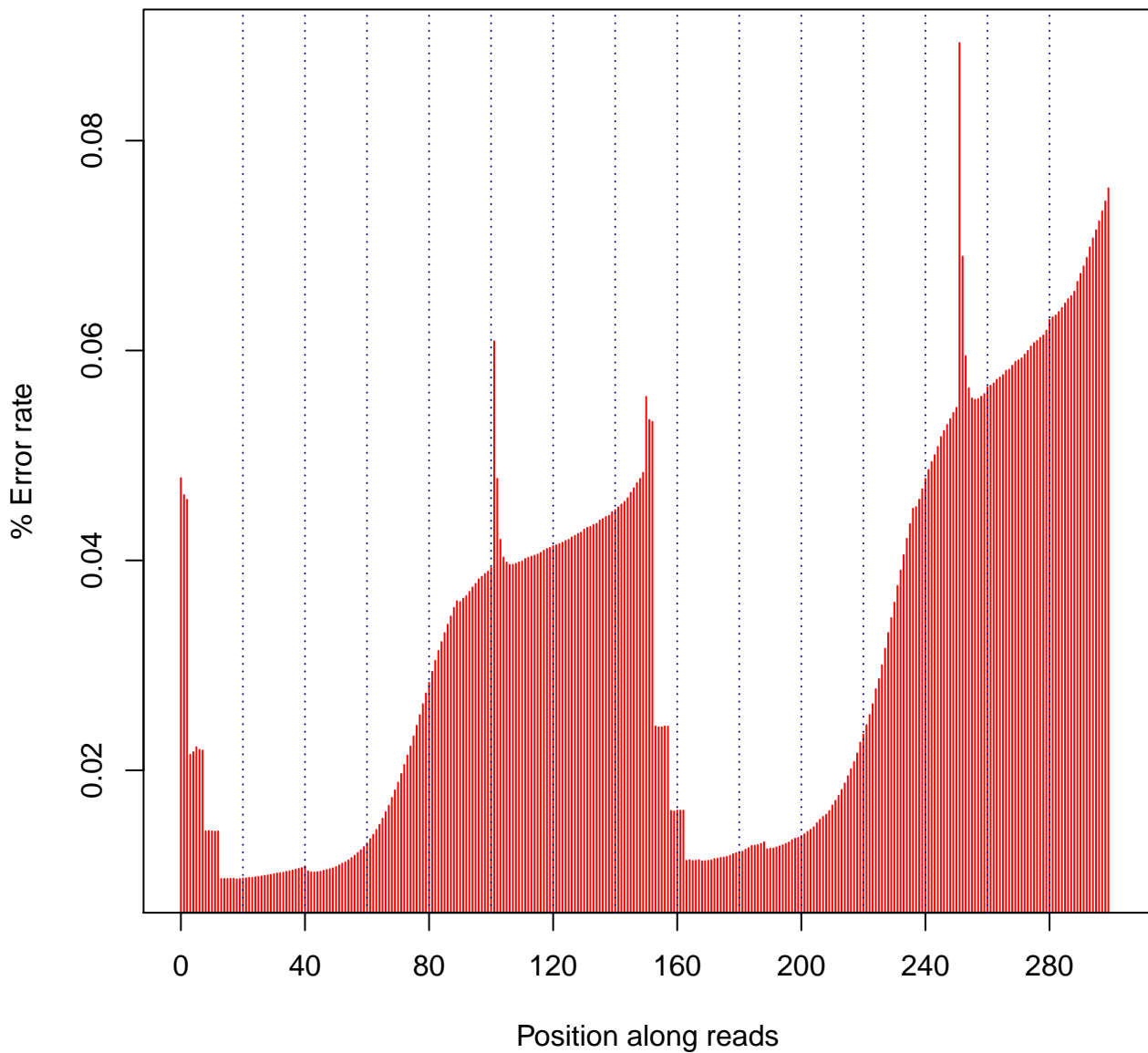

Supplement: Figure S2 — Distribution of sequencing error rate. (PDF) [file pone.0095458.s002.pdf]

# GC content–Coverage cutoff=\$cutoff

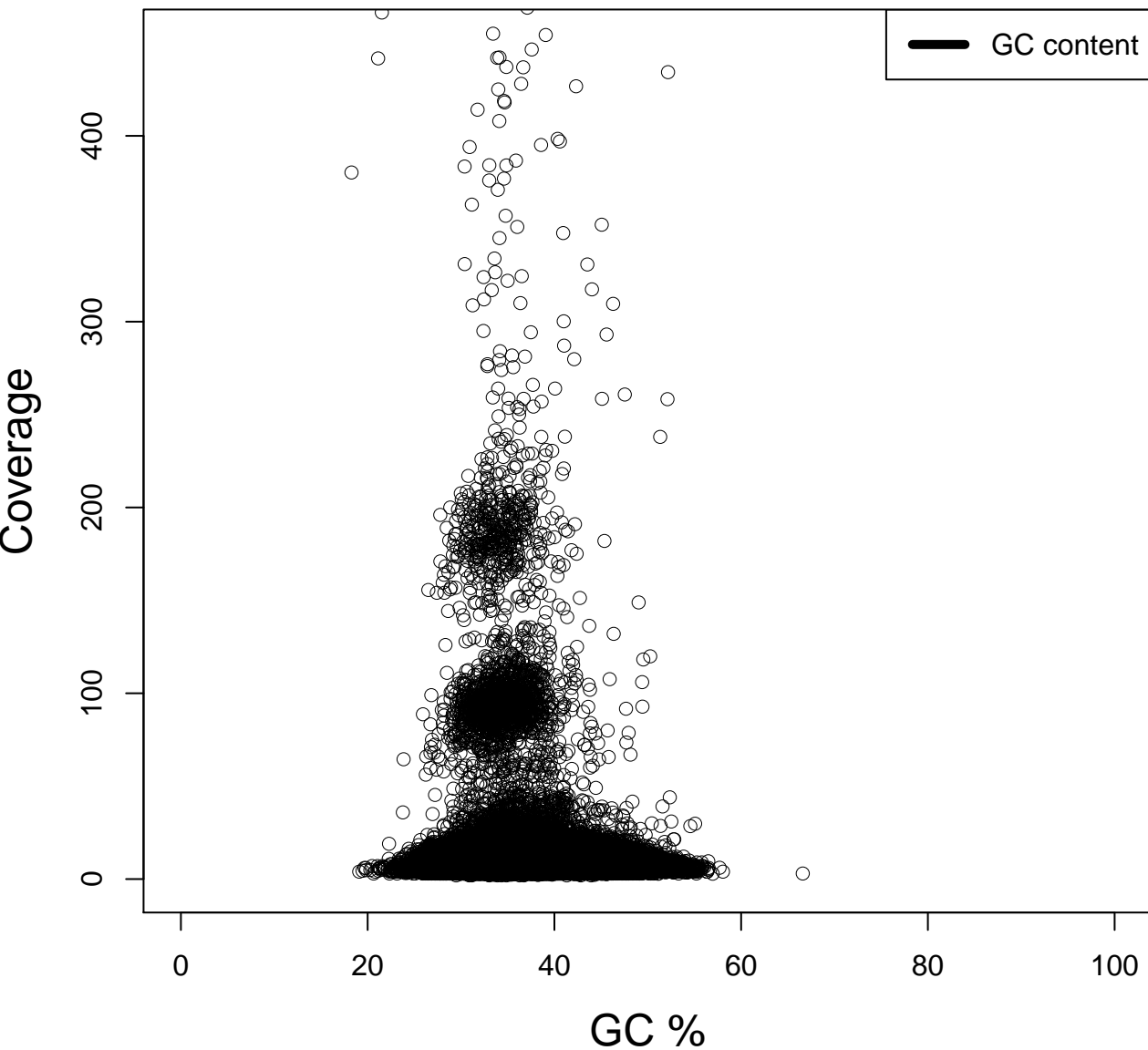

Supplement: Figure S3 — Distribution of GC content and contig depth. (PDF) [file pone.0095458.s003.pdf]
