# Supplementary material for: Rapid Development of Microsatellite Markers for Callosobruchus chinensis Using Illumina Paired-End Sequencing
Source: PLoS One. 2014 May 16;9(5):e95458. doi: 10.1371/journal.pone.0095458 (PMC4023940; doi:10.1371/journal.pone.0095458)
Supplement: Table S1 — Decontamination of valid sequencing data. (DOC) [file pone.0095458.s004.doc]

**Table S1.** Decontamination of valid sequencing data

| **Sample ID** | **Clean reads** | **Contaminated reads** | **Reserved reads** | **Reserved bases (bp)** |
| --- | --- | --- | --- | --- |
| Bpis1 | 46,596,993 | 1,543,401 | 45,053,592 | 13,516,077,600 |
| Bpis2 | 47,967,229 | 1,589,643 | 46,377,586 | 13,913,275,800 |
