# Supplementary material for: Rapid Development of Microsatellite Markers for Callosobruchus chinensis Using Illumina Paired-End Sequencing
Source: PLoS One. 2014 May 16;9(5):e95458. doi: 10.1371/journal.pone.0095458 (PMC4023940; doi:10.1371/journal.pone.0095458)
Supplement: Table S3 — Characteristics of 196 polymorphic SSR markers developed in Callosobruchus chinensis L. (DOC) [file pone.0095458.s006.doc]

**Table S3.** Characteristics of 196 polymorphic SSR markers developed in *Callosobruchus chinensis* L. (F=forward primer, R=reverse primer, Size = size of cloned allele, Ta = annealing temperature).

|  | **Primer** | **SSR motif** | **F (5’– 3’)** | **R (5’– 3’)** | **Size**  **(bp)** | ***Ta* (℃)** |
| --- | --- | --- | --- | --- | --- | --- |
| CCM1 | | AACA | TTGGTGCTAACGTACATGAAATGAA | CTCGCATAACGACTTCATGGATTT | 155 | 58 |
| CCM2 | | TAT | CAGGACTGGACTGGACATGTGATA | TGCTCAGCCTAATTCAACCTGTTT | 156 | 55 |
| CCM3 | | CA | AGAGTCGTCCTACGTTCGATTCAG | TTTCTGTGCTTTTGCTGACTGTTG | 110 | 58 |
| CCM4 | | ATA | CTGAACCGAATTTGAGCCGTAATA | CATTCAGATGTTTCCATCTGCTTCT | 133 | 55 |
| CCM5 | | TACA | CCGTTGTAAATCGCACTCAAAGAT | ACACTGGTCTGGTCAAATAGTCCC | 106 | 55 |
| CCM6 | | TTA | TGTAGGAGTAGCCATTGGGCTAAA | TCTGGAAGTCATGAATTGAAATCG | 132 | 55 |
| CCM7 | | TAT | ATAGCTGAACTTCGCCTTTTCATC | GAATTTCGATGACATTTTTGTCCC | 156 | 50 |
| CCM8 | | TTC | CCAACTGCAAACGTTGTTTTCTCT | TGTAGGGTTCCAATTGCTGAATTT | 90 | 55 |
| CCM9 | | TC | TTAATGTCTCTCCCTGTTTCTCCC | AGAGAGACAGAGATAATAGCGCGG | 87 | 55 |
| CCM10 | | TC | TCTCGCTCTTTATGTTCTACCTTTCT | AGAGACAGAGAGTGACAAAGCGAT | 144 | 55 |
| CCM11 | | TAG | ACCCTTTTCTGGGTAACGTTTGAC | TGCTGGAAAATGTGAAGAAGTCTG | 159 | 55 |
| CCM12 | | TAGT | CAGATTATGTTTATGAACACTCAGCATT | TAGCCACACACCAGTTTTATGTGA | 138 | 55 |
| CCM13 | | AT | TCTTTGTTTTTGTTGATTTTTGAAGGA | ACAACCGAACGAGTTTAATGCAAG | 138 | 50 |
| CCM14 | | ATT | AAAAGGCAAAAGGGTAAGGACAAG | AAGAAGGAAACAACATATGGTGACAA | 83 | 56 |
| CCM15 | | TC | CTCTCTGACTCCCCCAAACTCTCT | AGTGACAGAGAGATCGCTTTCGAG | 158 | 55 |
| CCM16 | | TTA | GAAATACGAGCATTCGGTCAAAAT | CCTTCGAGGTGGCTCTATTTGTAT | 104 | 55 |
| CCM17 | | ATA | ATTTCATGTAAGCCCTGCATTGTT | CGGACTTCGCCATTTGTGTATT | 131 | 55 |
| CCM18 | | CA | AATAATCTGTGGCAGATGGACTCG | CGCCAGAAAAACCATAACAAAAAC | 137 | 55 |
| CCM19 | | GTA | AGCTATCCTCATAATTGCTCACGG | CCTCCTTATAAGTGCATTTGTTTGC | 138 | 56 |
| CCM20 | | ATG | TGCCTATTGAGAAATTGGTACTCCA | GCAGTACATTTCAAACTACAAAAGCCA | 116 | 57 |
| CCM21 | | GCT | TCTGCTATACTTCCGATGGTTGC | CGTCTATACCAGACCCCTTGTGTT | 160 | 55 |
| CCM22 | | AG | TCCGGTAGAATTTAATTAGGCGTG | GTGAAGACTTACGGTGTACAGGCA | 85 | 55 |
| CCM23 | | AC | TTATTTACTGTTATCCGCAATCGCT | CATTTGTCGCAATAGCTTCAGAAA | 141 | 55 |
| CCM24 | | ATT | GTCTGTCAAACGTCAGAAGTGTCAA | ATTTGGCGGAAGTCAAGAGAAAAT | 90 | 56 |
| CCM25 | | TTA | AAACTCTTGCGTTAATTGTGCCAT | TTTACTTGATGACTTGGCAGAGCC | 99 | 55 |
| CCM26 | | TAT | AATTTCTCTCGCATGGTTGAAGTG | GCCTGGACGAAAAAGTACACAAAA | 142 | 56 |
| CCM27 | | ATA | TGTGTTCTATCTAGCGTGACTGGG | ATGCTTGTACATTTTGACTTGCCA | 158 | 55 |
| CCM28 | | ATTT | ATTCAATCTGGTTGGTTTCGATGT | ATGTAATGCCACCCTGTAATTGG | 112 | 55 |
| CCM29 | | TA | CAATGGCATGTCCACCACTACT | GATAAGCAATTGCCTCTTATCCCA | 146 | 55 |
| CCM30 | | TG | TGCAAGATTATTTCCACCAATGTG | GATGTTTCAATGCGAGCCTAAGAT | 160 | 55 |
| CCM31 | | CTC | ACTGTTCATACTCCTCCTCCCCTC | TGGAGAAGAAAAATAGCAGCAAGAG | 115 | 55 |
| CCM32 | | AG | TGCAAACTGAAATTTGAGAACCAA | TGAAACAACCAAAATTTTAGACTCAAGA | 132 | 55 |
| CCM33 | | ATA | ACGACTTTATCCACAGAAGCCAAT | TAATATTCATGGACTTGGAGGCGT | 141 | 55 |
| CCM34 | | TA | CTGGCTGTTATTTTTCCAAAGCAC | TTGATTGGTTTTGTCAAATGATTCAC | 117 | 55 |
| CCM35 | | AAT | GCCTGACACCAAACACATTTACAC | GGCATTTTTGTTTCCGTTTCTTTA | 156 | 55 |
| CCM36 | | TTAGG | GGTGAGGTTAGGTTAGTTTAGGTGAGG | GCTAAACAAACACTGCCACGTACTA | 132 | 55 |
| CCM37 | | ACA | GCATCTCTAAGTTATCGCCACTCA | ATATCCTGATGGTTGACGGGTTTA | 133 | 56 |
| CCM38 | | ATA | CCAATTTGGAACGAGAGGCTACTA | AATTGGTCTCCATGGATGAAATGA | 142 | 56 |
| CCM39 | | TCTT | CTTTCTTGCATTTTTATTTGTTTCTTCA | AAGCAGAGAGGAAGAAAGGGAGAG | 105 | 55 |
| CCM40 | | TAT | AGGGGATATCGTGTTCTCTGTTCA | GATCGAACCTCGAAGCAGAAGTT | 115 | 55 |
| CCM41 | | CA | TTGTTGCATGAAAATTTTGAAAGG | AAACATTTCTTGCATATGTCCGCT | 116 | 55 |
| CCM42 | | GTA | AGTGCGAACTAAAGCTTTCCAATG | ATTACAATGTGTGACAAGCATCGC | 135 | 55 |
| CCM43 | | TCCT | AAGTCTTGAGACCTTCCTTCCGTT | AAAAAGGTTGGGAGAGAGGATGG | 82 | 58 |
| CCM44 | | AGT | GGAAACAGAGATACCTTCAAGGAACA | TAGTTTTATGGTTCTGTGGGGTCG | 137 | 55 |
| CCM45 | | TCA | AAAATGCCTTCCCAATTTTCTGAT | TTAAATAGAGAACCCTGGTAAGCGG | 146 | 57 |
| CCM46 | | AGAC | AACGGACAGACAGAGAGATAGTCAA | TCTGTTTGTTTGCCTGTTTGC | 134 | 55 |
| CCM47 | | TGTA | ACACCTCAATTCACATAACGTTGG | TGTAAATCATGCTCAAAGATTCCG | 137 | 57 |
| CCM48 | | GAAG | AAGGAACGACGAGAAGGAGGAATA | CCTGCCTTCCTTGCTTCCTATC | 136 | 55 |
| CCM49 | | TATC | GTTGACCGTTGGACAATAAAGCTA | TGTCACTACAATCGATTCGTCTCAT | 145 | 55 |
| CCM50 | | TACA | TATCAAAAATCATGAATCCTGGCG | ACACCCACATCTGTCAGGAAATCT | 139 | 58 |
| CCM51 | | TTC | GTTCTAAGCTCGGTTCACGAGGTA | GTTAGTTGCATATCGTGCAGCATT | 83 | 55 |
| CCM52 | | TAT | CGCCTTTTCATTATCTCAGAATTGTAGT | CGTGTCGATGATGTGAAGAGAAAA | 160 | 57 |
| CCM53 | | TAA | TTACCATGCAGCGATCATACTGAC | TGGCTAAACTTCGCCTTTTCATTA | 124 | 55 |
| CCM54 | | TC | TCCCTTTCCCTCTCTCTCTCTCTC | TGACATGAATAGTCCACAAATCAAAGA | 129 | 55 |
| CCM55 | | CAT | GACGGAGTTGCTCTGAGACCTTTA | TGGGTACCTAATGGTTACACTGGC | 115 | 56 |
| CCM56 | | TG | CCTAAGAGTTTCGTTTTTGCATGG | GGCAAATTGAGCTCAGCATTAGAT | 110 | 55 |
| CCM57 | | AG | GAGGACAATGCAATAAACAAGCAA | TACTGTTGCACTTGGTATGCACG | 98 | 57 |
| CCM58 | | GA | GAGAGAGAGAGAGAGAGGCAGGGT | CGTTGCAAGGGAGAAGTAGTCAGT | 143 | 58 |
| CCM59 | | TAA | CGTGAATAGTGTACAACAAAAGCTCA | AATAGCCTGTGACTAGATTTCGCC | 129 | 55 |
| CCM60 | | TA | ACAGCGGCCTATGGTCTATTACAA | AGTCCGGTTGCAACTATACAGTCC | 91 | 56 |
| CCM61 | | AAT | ACACGTCAATCAAGAAATGGATCA | ACGTCCTACAGGCTGACAAGAGAT | 116 | 55 |
| CCM62 | | TACA | TTAGAAACCCTTGTAAATCGGGCT | CTTCTTTGTGGCTGGAACATCTTT | 120 | 55 |
| CCM63 | | CT | CCCCAATGTCTCTCGGTCAC | GGAGAGAGAAAAAGAGACAGAGATCG | 132 | 55 |
| CCM64 | | CT | CTCTCTCTGTCTTTCGCTCCC | GACGAGAGATGAGAATGAGAATGAA | 147 | 55 |
| CCM65 | | TA | GTACCGCGGGTACCTACGTACTTT | GTGTTTACCGTTTTCAAACGCAG | 81 | 55 |
| CCM66 | | AAT | GGGCTAATCGCACAACTGTAAATC | AAATAGCCAGGAGCTAGACTTCGC | 141 | 58 |
| CCM67 | | TTAC | AATACAAATGCGATACCAGCATCA | GCTTAAGGGATTCCTATACGTCCA | 157 | 52 |
| CCM68 | | ATA | GACACCTGAGCAGTGATTTGATGA | AACTCCGCCATTTGAAGATTTGTA | 80 | 55 |
| CCM69 | | AATA | CAACCGTTCTGAAACATAGGTTCG | CAAACTGTACCTTTCCATCCTTGG | 101 | 58 |
| CCM70 | | AG | CACCGATTCTGATTAGTAGGTGCC | GACGATTTGATTGAGCCTTTGTTC | 129 | 58 |
| CCM71 | | CGA | GCACTATACTCAGGACGTCTCGC | CGACGTCTCATCTCTTCCAGGTAT | 132 | 55 |
| CCM72 | | TAA | TGCATATGTCCAAGAACAATCTCG | ACTCTACATCCCCTGGAGCGTTAT | 133 | 55 |
| CCM73 | | ATT | TTTCAGACCGTTAATTGGTGAACA | GGGTATGAAGATATGAGGCTCCAA | 157 | 50 |
| CCM74 | | TAGA | GCGTTTTTATTGGGAAACCCTTTA | GTAATTGGGTGCGTTTGTACCTGT | 104 | 50 |
| CCM75 | | ATG | ATCTTTGTTGATTTGCGGTTGTTT | TCACTAATCTTGTTTAGGAAGCAGGAA | 131 | 50 |
| CCM76 | | AG | TCTAAAGCAGCCTTAAGTGGAGGA | TCTCAGCCACCTTTCAGGATTTTA | 118 | 50 |
| CCM77 | | ATAG | AACGACGTTTTGATGCTTGTCATA | TTCCCATCTTATGAAACACCCTGT | 120 | 55 |
| CCM78 | | ATT | CAAATGCACTGTACAACCACTTCC | CTTAAGGCTGAGCGGCAACTATTA | 158 | 55 |
| CCM79 | | ATA | AATAAGCCCCAGTTTCAACGATTT | GCATAAAAGTCAGTGGAGGAGCAG | 159 | 55 |
| CCM80 | | TTA | TGAGTAGCCACAGACTTCACTTCG | TCGTGTCTGATGCTCTAGAACTGG | 137 | 55 |
| CCM81 | | TTA | ATTGCTGCGTAACAATAAAGGCAT | TATAACCAGACCCGGAAATCACAT | 116 | 55 |
| CCM82 | | AT | TACATGGAGGTAAATCCGTTCTGC | TTTAAGTACCCGGTGCAGTTTGTT | 113 | 55 |
| CCM83 | | AAT | AGGGTTTTTCCCGTTTAACGATATT | CGGATCTGGAAGCAGTTTTGTTTA | 140 | 57 |
| CCM84 | | TTA | ACTCCTCGGCTAGCAGTGACCT | TCCTGATACAAGCATAATAAGCGGA | 159 | 55 |
| CCM85 | | TTA | TGCAGCACATGGTTAAGTGTAAGAA | CCAACAGGCTTCCATTTATAGGAC | 159 | 55 |
| CCM86 | | TCT | GGTAAAAGGTTGGATTTCGCCTAC | TTAATCACTTTGCTTCGACTGCAT | 158 | 55 |
| CCM87 | | TAC | AATTCGTTATTGGAAGCTGTTGGA | AGAGGAGATCACCCACTTGTAACG | 144 | 55 |
| CCM88 | | CACCT | AAGCACTAGGAAGTGATTGGAGGA | TGTCTCCTTTTGTCCGCATTTATT | 157 | 55 |
| CCM89 | | AAT | AAGGTAGCCTAAAGGCAACACTGAT | AAAGATCCTAAAAATCCTAGTTCGGG | 81 | 56 |
| CCM90 | | AG | ATGCTCGCGAGAGAGAGTGC | TCTCACCCTTCTTCTCTTTCTCTTTCT | 160 | 56 |
| CCM91 | | TATC | CCTTTGTAGGTCTGAATGGATGAAC | GGGAGCATTGTTCCAATTATTGAC | 146 | 56 |
| CCM92 | | AAT | CCACCATCAGGTTTGGAAACTTAC | TCCGCCATTTGAAGATTTTTGTAT | 99 | 56 |
| CCM93 | | TGT | AATATTGTCGAAGCGGAGTCTGTC | GTACTACTTCCCCGTCGGAACAA | 148 | 55 |
| CCM94 | | ATA | TAGGAGGAGGAAGAGGACGTTAGG | ATCATCTGTGTGTCCGTGTTCAAT | 108 | 55 |
| CCM95 | | CAA | ATGAGTAATCTGCAATGTCCGGTT | TGAATACAGTTGTGTTTGCCATAGG | 157 | 55 |
| CCM96 | | TAC | CTTGCATGGCATCATCATTTTAAG | AGTTGGGCGTCTACTGTGATTTGT | 102 | 55 |
| CCM97 | | AC | ACCCAATAACAGGAAATGCAAAAA | ACTGGGTGATTCCGTGTAAGTTTG | 127 | 57 |
| CCM98 | | ATA | AACTTTAGCCGATTTCGGTTTTGT | TATAACTGGATTTCTCTGCTGGGC | 140 | 55 |
| CCM99 | | GTA | AATGCACAGCATTTGTAAGGTCTC | TGTTTACTCATCGCAAACTAAGCG | 97 | 55 |
| CCM100 | | TAA | TTCGACACAGAGAATGGATCTTCTT | GTAGGAGTAGCCGTTGTGGCTAAA | 156 | 55 |
| CCM101 | | CCA | TCTGTAAAATGCTAACCTGGTGCT | AAAATATTATCTCAAAAGGGTGGGG | 105 | 55 |
| CCM102 | | CCTAA | ACGTTCTAGCCTGTGGCTACTCAA | AATGGCCTGACTCACACTCTTCTC | 140 | 58 |
| CCM103 | | TTA | TCAACATCGTGTCTTTGCTGATTT | CTTGGACTTTATAGCTTCTGCTCCA | 84 | 55 |
| CCM104 | | TATT | GTTACATATAAATGCGGCCACCTC | TGATAGTAAATGGTTGTAGTGTTGGTGG | 109 | 55 |
| CCM105 | | TG | GTGTGTCGCAAGTTTTCGTACATT | TTCGAGGTGTGAGAAACTCAAGTG | 131 | 55 |
| CCM106 | | AATC | TCGATTATTGTTACCGTGCTCTCA | TCAGGGACTGATGCTAGGAGATTT | 142 | 55 |
| CCM107 | | TTA | GCTAAACTTCGCCTTATTGCTTTG | CGGAAACTCCATTTTTACAAATATTACA | 151 | 55 |
| CCM108 | | TGTA | ACTTAAATTACCCGGTGACGTGAA | AACTTTTGTAAATCGCGCTCAAAG | 131 | 55 |
| CCM109 | | TTG | ATTTCTAATGAACACCGCGATCAG | AATAGGCTTTCGATTGCAGACAGT | 158 | 56 |
| CCM110 | | TAA | GCTTCCAAATATCACCTCTACCCC | GGTGTATCATCTGTGTGTCCGTGT | 120 | 55 |
| CCM111 | | TAA | GAGTGAAAATCAATAATACGTACAAGGG | TTGACATTACCTTCATTATCCCAAAA | 117 | 55 |
| CCM112 | | AC | CATTTTACAGATGGCATTGTTCCC | TCTTGGCAAAAAGTATTCACCACA | 126 | 55 |
| CCM113 | | ATAG | AAGCTATGATAGACTCGCTTAATCCG | CACGAACCTAATTCAGAGAATTGTGA | 159 | 55 |
| CCM114 | | TAGA | TTCTTGCAGTATTTTTGAACACGC | CACCAGTTTTTGGGTTATACCGAG | 150 | 55 |
| CCM115 | | TAG | CACAACGACCAAGCTACTATGCAC | ATGATAGTAGACGCCAAGAGGCTG | 118 | 57 |
| CCM116 | | TAA | AGCACTTGTGCTACACTCAACTCG | AGGTTCTCCTATATGTATCGCGCA | 123 | 56 |
| CCM117 | | TAGA | CTCGAGGCTACTCCACCTTCAC | GGGACTAGCAGTTGCTGGTAAGAA | 129 | 55 |
| CCM118 | | TGT | TTAATTCTGGTCCTGTTCCGTTTT | TCTTCTAATCCCATGACTAGCTTCAC | 130 | 55 |
| CCM119 | | ATA | TGTCCGTATGATTATTGCGAATTG | TATAATTCCTCTGCCCCCTTGTTT | 151 | 57 |
| CCM120 | | AAT | CAGCTTAAGTGTGGAGCGTTATGA | TGTGATTGTTTGTGATTCTGAGCA | 148 | 55 |
| CCM121 | | AATA | TGGATATGTCTGTAAGCATCCCAA | GCTCAGCGTTCTAGTGACTTCAGA | 80 | 55 |
| CCM122 | | AATA | TGAGAATTCCCAAAATTACACCCTT | ACAGTTTCGTGTTTTCGAATTGGT | 110 | 58 |
| CCM123 | | ATA | TTCCTTTTCTCTCCAAACAATGCT | AATATTTTTGTTCGGAGGGGTGTC | 150 | 55 |
| CCM124 | | GAA | CCAAGAACATTGGCAATACTTGAA | TAGGTTCTTGACCTTCGTTTCTGG | 130 | 55 |
| CCM125 | | GAA | AGCGTGTTAAAAAGAGAGTGGACG | TATTATTCTGCAGATGTGCCGCT | 144 | 57 |
| CCM126 | | ATT | TTTTGTATGCATCAATTAACGAGTGA | TTGTTGTTGTTCTCTTCGTTGGAC | 153 | 55 |
| CCM127 | | TCAA | AAGGAACACCAAGCCTGATCAATA | GCATGACCATCTCTGATACCTTGA | 135 | 55 |
| CCM128 | | TACA | TCCTTGAGACGTATGCAAAGTTGA | AATGCAATAAATGGCTCTTTTCCA | 160 | 55 |
| CCM129 | | TAA | ACGTGTTTGTGATCTACACGCATT | GGTTTTCGGGCATTTTAGAGATTC | 141 | 55 |
| CCM130 | | TC | GAATCTCTAAAATGCCCGAAAACC | AGGAAAATAAGTGGACCCAGGAGT | 158 | 55 |
| CCM131 | | TCTA | TGCGGATCACCAACTAAAATACCT | TAAGGCTAACTGCATATTTTGCCC | 84 | 55 |
| CCM132 | | CTA | TAAGAACTTTGCCATCTCCCAGTC | AAAGCACGCATACAAGAATCTGAA | 126 | 55 |
| CCM133 | | TTTA | TACAACTGTAAATGGCGTTTGGC | ACAATGCACAGTGTACGACGAGTT | 95 | 55 |
| CCM134 | | TCT | GTCATCATCGCTGTGTTTGTTGTC | TGGAGAGGATCAGAGTAGACCTCG | 136 | 57 |
| CCM135 | | GTA | AACTGACAAAAATACATTGCAGCG | GTGATCACTAAAACCCCTCAGACG | 129 | 57 |
| CCM136 | | TAGA | GTTAAATCACAAGATCTCGGTGGC | ATTGGGTGATTTACCTGTTGGAAA | 89 | 55 |
| CCM137 | | GAT | TTTGAAGTGACAGCTCGTGTATCC | AAATACGGTTTGTTTCTGAATCCG | 150 | 55 |
| CCM138 | | TG | CCGTATTTATTGGCTAAGGCTTCC | ACCATCACGGGTAAATGATGACTC | 154 | 55 |
| CCM139 | | TAT | ACTGCTGCCTTAAAAAGAAAGGCT | AAGGAACGGTTAGCACTTGGATG | 141 | 55 |
| CCM140 | | ATAG | TCTACGCATGCAGATTTTTGTGTT | TCCTGTACATATTTCAGGTGCATTACA | 105 | 55 |
| CCM141 | | AATA | AAATTGGTTTATCTGTTGGTGGTG | CTTTTCTGAGGAAATCACGGAAAT | 151 | 55 |
| CCM142 | | TC | TAAATGTTATCGTTCGCGCTTTTT | TGTGCATATTTTTCCGTTTGTGTC | 95 | 58 |
| CCM143 | | TAA | TTCGAAGAATTCTAAGCGTATGTGC | CGGTTGAGTAGAAGTAGCCGTTGT | 133 | 55 |
| CCM144 | | ATC | GCCTGGTCAGATCAATCAAATACC | ACCCATTCGTGATGTCAATACCTC | 136 | 57 |
| CCM145 | | TAA | TGACATCACGAATGGGTAAAGAGA | TGCCAAGTGTATTATTTTGTATCTGGG | 138 | 57 |
| CCM146 | | AAT | GATCACTGTTCCCATGACAGGATA | ACATTCGCCGAGTTGAAAATAAAG | 117 | 55 |
| CCM147 | | TACA | TTAGAAACCGTTGTAAATTGCGCT | GTGGTTTCGTTCACTTTTGAGAGG | 132 | 55 |
| CCM148 | | CTAT | TGCAGGAACCTAATTTGTGACTGT | CAGCTACCAGATCACGAAGAGTTG | 129 | 55 |
| CCM149 | | TTC | CTTTGCAGTTACGAGAGACAGGGT | TGCCAACCTTCTATAAGAACACGG | 139 | 57 |
| CCM150 | | TCA | CGGCTTCAACTATACGGTGGTTAT | TATTTTCCTATTGGCCCCCATATC | 136 | 57 |
| CCM151 | | TTAT | GATAAGCGGTTAAAACAGTTTACCAGT | TGGAATTGTAGATGTATTTGGGTCTT | 155 | 55 |
| CCM152 | | TA | GCGATCCAATTTGCCATTCTAA | GAGCTCAAAAATCAGGCCCTTTAT | 140 | 55 |
| CCM153 | | TC | TGCACAGTGGTCGTTGTTTAGTCT | AATCTATCTTCCCATGCATTCCCT | 159 | 58 |
| CCM154 | | TAC | TTGATCCTTTTCCTCCCAATAAAA | TTCGTCCTCATATAGCACAAATGTT | 145 | 53 |
| CCM155 | | TAA | CAGCTTGTAGCTGAACTTTGCTGT | ATGGGTTTTAACGAATTATGTGGG | 119 | 55 |
| CCM156 | | TAG | TTGATAGTTGCCCCAACTCCTAGA | AGTCGAATGTTGCCTACTAGCCTG | 83 | 55 |
| CCM157 | | TGT | TGTATCGATTGGAGGCCAGATTAT | ATTACTTCCAGAACGCACCCTGTA | 153 | 55 |
| CCM158 | | TAT | CATGGTTGCCACAGAAACAATAAA | TTGTACGGTCCTGCCTAAATTCAC | 94 | 55 |
| CCM159 | | TAT | TGTTAGGCCTGAGACGGACATAAT | AGATCCAGGAGCTTTTCCCAATAC | 146 | 55 |
| CCM160 | | TCT | AGATGCCTTGATGTAAGATGGGAA | ATTCAGGAAGTCGCCAGGAATAG | 136 | 55 |
| CCM161 | | GCA | AAGGAACTGCAAGTTTTCTTGACG | AACTCACTGCAAAAACATTTTCATCA | 101 | 55 |
| CCM162 | | TACA | GTAAATCGCGCTCAAAGATTCAGT | TCAACACAAACATGCATCAAAAGA | 113 | 56 |
| CCM163 | | GCT | AAAAATTGGCGTGATTAAAAAGGG | GCACTAGTATCTCTTTCCGAACCG | 88 | 56 |
| CCM164 | | TAA | TGTTCGATTTTGCAGCTAACTTACA | ATGGGCAACCTGATTCAACAGT | 160 | 55 |
| CCM165 | | AAT | CTTGTACGCTTGTTGAAGTCAAGG | GTGGACAAGGATCAAAATGGTCTC | 107 | 58 |
| CCM166 | | TAT | TAAAATTTATCTCGCATGGTTGCC | TTGTACGGTCCTGCCTAAATTCAC | 109 | 58 |
| CCM167 | | TAT | TGTTAGGCCTGAGACGGACATAAT | CACGTCACACTAAACATAGCCCAG | 96 | 58 |
| CCM168 | | ATA | ATAGGTTGTGGGAGTCTGAAGGTG | AGTGGTCAAGAAGGACTATGTCGG | 138 | 55 |
| CCM169 | | TAT | CAGAAGTTGAGACATACAGTGTCGG | TCATTCAGAGAATAATAACAGCGGC | 160 | 56 |
| CCM170 | | ATT | GGGTGTGAACCTGTTGGAAATAAA | AGAGCTAGACCCAGGGTAGCAAAT | 125 | 56 |
| CCM171 | | AG | GAGCAAAAGAGAGAGAGAGACGGA | CTCTTGATTGACGCATTTCTCCTT | 144 | 56 |
| CCM172 | | TTA | GGAGGGTAAGATGAGTAGCCAGGT | ACTAAGAGGAGGAGAGAGGTGGGA | 159 | 56 |
| CCM173 | | TTA | TGAGTAGCCAGGAGCTAAACTTCG | GGAGGCTTGGGAGAGATATGTTTT | 128 | 56 |
| CCM174 | | GACA | CGGTAGACCAACTAACAAACTTACCG | CTGTCGGTTTATCTGTCTATCGGC | 140 | 56 |
| CCM175 | | AAAT | CGTTTGTGTTTTCTTTTTCTTCTACCA | GACAAAACAACGCCACAGATAAAA | 142 | 56 |
| CCM176 | | CTA | TCTGTTCTTTTCAAGGAGCAACAT | CATTCGATAATGATAGAAGTGAGAACAA | 138 | 53 |
| CCM177 | | ATAG | CGGTTGAGTAGGAGTAGCCATTGT | TGCAGGAACCTAATTTGTGACTGT | 149 | 55 |
| CCM178 | | TAT | GGTTGCGGTAGAAACAGGTAGGTA | GCCTGGTCGAGAAGTTACAGAAAG | 154 | 55 |
| CCM179 | | CTA | TAAGAACTTTGCCATCTCCCAGTC | AAAGCACGCATACAAGAATCTGAA | 123 | 55 |
| CCM180 | | TTTA | TACAACTGTAAATGGCGTTTGGCT | GGCATTTCAACAAAAGTACGCTTC | 116 | 55 |
| CCM181 | | GCA | GTCATGTCAGCAGAGGGAAGGTAG | AATCCCGAGCACGTCCTTATAAAT | 149 | 55 |
| CCM182 | | AAAT | TTTCCTATGATTGGGTGTGAACCT | AAATTTGTGAAATGTCTGCTTGGG | 139 | 55 |
| CCM183 | | TCTT | ATTAAGTCGTTCCTGCTTCTGCTC | AGAGTTAAAGATCAGATGTGCGGG | 86 | 56 |
| CCM184 | | ATT | ATCCAGTGACTACTCCTCACACGG | TGGCACTGTTAAACATGTTCTCGT | 153 | 56 |
| CCM185 | | AAAT | TTGTCTCTGTGATAGTCCCACCAG | TCTATAACTCTCCGTGGGCAGAAC | 134 | 57 |
| CCM186 | | CA | CTTTTTGTTGTTTCCTAATGCGCT | ATTCGGCGGAAAATTTTACATACC | 117 | 57 |
| CCM187 | | TAG | AAACTTTCCTCTATCACGCCCTTC | CCAGTGACATTTTAGTCATTTGCG | 105 | 55 |
| CCM188 | | ACT | TTGGTTTAAGAGCCAATGGAAGAA | GTGAGGCTCTCTTTTGAAACCTTG | 155 | 55 |
| CCM189 | | AGT | GGAAGTTGCATATGTGAAGTCCAA | AGGATGCATTCTGTGTCATCTGTT | 107 | 56 |
| CCM190 | | ATA | ACGTCATGTACCTAGCATGGGATT | CGCATTTTGTATTGCATTGTCCTA | 91 | 55 |
| CCM191 | | ATT | TGCGTTCAGATCCTTCTTTTTGAG | TCAACAATAATGCTTGGGTTTTCC | 160 | 55 |
| CCM192 | | CAG | CGACGTATTATCTAAAAGCCTGCG | ATGTTTTTCCTGATAACAATGCCG | 93 | 58 |
| CCM193 | | GAA | CTCTGACTATGATAGCGCTGGTGA | ACTGATTGATCAAACTAGCAGGGC | 144 | 55 |
| CCM194 | | GA | AAAGAGAGAGTGAGAGGGAGACCG | TTAAGAGCGCCAATTTTCTTACCA | 106 | 53 |
| CCM195 | | GA | AGCAAGAGACAGAGCGAGAGAGAG | CGAGCGAGAGAGATTAAGGAGAGA | 150 | 60 |
| CCM196 | | TCCT | CTACTTCCTCCCCTTTGCTCATCT | TTGGATACGATCTTGTCTGTCGAA | 118 | 55 |
